# P-876. Decision Analysis of Short versus Ultra-Long Course Antibiotics for Complicated Intraabdominal Infection

**DOI:** 10.1093/ofid/ofaf695.1084

**Published:** 2026-01-11

**Authors:** Christopher A Guidry, Robert G Sawyer

**Affiliations:** University of Kansas Medical Center, Kansas City, KS; Western Michigan Homer Stryker M.D. School of Medicine, Kalamazoo, MI

## Abstract

**Background:**

Short course antibiotic therapy (4 days) following source control for intraabdominal infections has become standard of care. However, recent data suggests that ultra-long course durations (28 days) after source control may drastically reduce recurrence rates. A randomized trial of ultra-long course therapy for complicated intraabdominal infections is currently enrolling. The purpose of this study is to evaluate these two treatment strategies using a decision analysis model.

**Methods:**

A decision tree was created from the perspective of a healthcare administrator. The model evaluates the rates of recurrence between these two treatment strategies as well as potential negative impacts of overtreatment and per-day risk of antibiotic-associated adverse events. Model values were taken from the published literature or estimated by the authors. Base-case and probabilistic sensitivity analyses were performed. The model was created using TreeAge Pro Healthcare (TreeAge Software, Inc., Williamstown, MA).

**Results:**

On base-case analysis, short course therapy maximized utility with an overall utility of 0.97 versus 0.92 for ultra-long course therapy. Probabilistic sensitivity analysis, after 100,000 iterations identified short course therapy as the optimal strategy for >99% of model iterations.

**Conclusion:**

In this exploratory model, short course therapy resulted in higher overall utility values compared to ultra-long course treatment. Further work is needed to confirm these findings, including completion of the currently enrolling EXTEND trial.

**Disclosures:**

All Authors: No reported disclosures

